# Effect of the dual endothelin receptor antagonist bosentan on untreatable skin ulcers in a patient with diabetes: a case report

**DOI:** 10.1186/1752-1947-5-151

**Published:** 2011-04-16

**Authors:** Fátima Álvarez Reyes, Cristina Luna Gómez, Manuel Brito Suárez

**Affiliations:** 1 Service of Rheumatology, Hospital Universitario Nuestra Señora de la Candelaria, ES-38010 Santa Cruz de Tenerife, Spain

## Abstract

**Introduction:**

Refractory skin ulcers are a major burden in patients with diabetes. Their pathogenesis is multifactorial, and data increasingly implicate endothelin as a mediator of diabetic macro- and microvasculopathy. Here we describe the first reported case of an endothelin receptor antagonist being used to successfully treat refractory skin ulcers in a patient with diabetes.

**Case presentation:**

An 85-year-old Caucasian man with a 30-year history of type 2 diabetes developed multiple skin ulcerations, including a right heel ulcer. Despite appropriate treatment, the ulcer showed little improvement and the risk of amputation was high. The patient was treated with the dual endothelin receptor antagonist bosentan. After three weeks of treatment, major improvements were observed, and after 21 weeks, all ulcers had healed. No abnormalities were observed during monitoring of blood pressure, erythrocyte sedimentation rate or serum aminotransferase levels.

**Conclusion:**

In patients with refractory ulceration associated with diabetes, bosentan may be of real benefit, especially in terms of amputation prevention. This case supports the proposed role for endothelin in the pathogenesis of skin ulceration in diabetes and is suggestive of a potential benefit of bosentan in this patient type. This case report is of interest to diabetologists and dermatologists.

## Introduction

Non-healing skin ulcers, particularly those affecting the heel, are major complications in diabetes and often lead to amputation below the knee [1,2]. The pathogenesis of these ulcerations is often multifactorial and includes Macrovasculopathy (arterial insufficiency), microvasculopathy (neuropathy and diabetic skin microangiopathy), and an increased propensity for infection [3]. Endothelin may underlie the development of vascular complications in diabetes, and there is increasing evidence supporting a role for endothelin early in the pathogenesis of diabetic micro- and Macrovasculopathy [4-6]. In this report, we describe the successful use of bosentan, an oral dual endothelin receptor antagonist [7], in a patient with diabetes who had multiple non-healing skin ulcers, including one that affected the heel.

## Case presentation

Our patient was an 85-year-old Caucasian man with a 30-year history of type 2 diabetes. Sixteen years after being diagnosed with hypertension following a stroke that caused mild residual right hemiparesis, he was successfully treated for prostate cancer with radiotherapy. Ten years later an electromyogram showed mixed peripheral polyneuropathy with axonal predominance.

While the patient was ambulatory, a 2 cm ulcer appeared on his right heel after prolonged exposure to heat, and it increased in size despite appropriate wound care and glycemic control. Less than three months later the patient was unable to walk and was subsequently admitted to our hospital for congestive heart failure.

**Figure 1:**
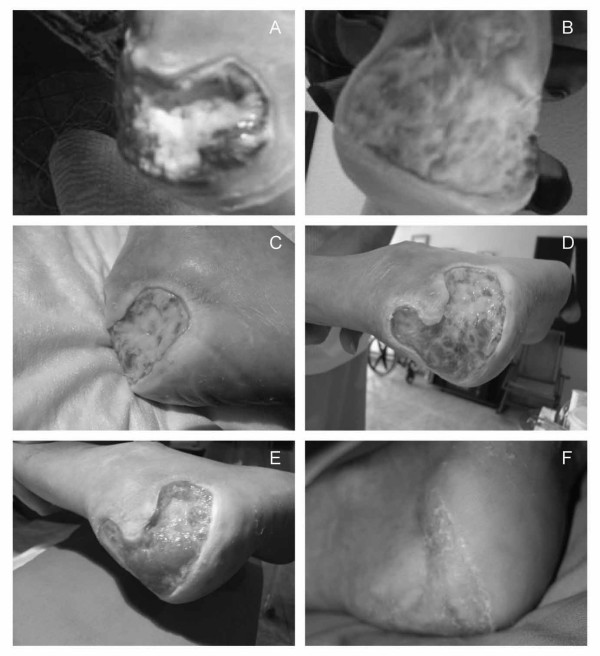
**Ulcer status at presentation, after standard therapy and following treatment with bosentan**. **(A and B) **Wagner grade III heel ulcer on admission showing exposure of the calcaneus with an inflammatory aspect and very limited granulation tissue covered by purulent, fetid exudates. **(C and D) **After three weeks of standard therapy, little improvement was observed, and after five months of conventional local and systemic therapy, the ulcer remained at Wagner grade III, affecting the total posterior face with partial exposure of the calcaneus but sparing the Achilles tendon. **(E) **Notable improvements were observed in the heel ulcer after the initiation of bosentan therapy and two weeks' treatment at a maintenance dose, with marked granulation tissue apparent on the heel. **(F) **Complete healing of the ulcer was observed after a total of 21 weeks of bosentan therapy.

A skin examination performed at the time of admission showed the heel ulcer to be extensive (Figures 1A and 1B), and it was evaluated as grade III according to the Wagner grading system [8]. Three additional decubital lesions had developed on the sacral area, external malleolus and flexure of the right ankle. These lesions were evaluated as Wagner grade II and were well delineated with a necrotic appearance, without granulation tissue, and covered by purulent exudates.

A general examination indicated that the patient had congestive heart failure probably due to ischemic heart disease. Echocardiography was not performed, and electrocardiography showed no evidence of myocardial infarction. The patient's diabetes was poorly controlled, with neutral protamine Hagedorn insulin measurements of 40 IU in the morning and 15 IU in the evening, and glycosylated hemoglobin levels >8%, which required adjustments of his insulin dose and addition of on-demand fast-acting insulin. He was also receiving bicalutamide 50 mg/day, candesartan 16 mg/day, furosemide 20 mg/day, triflusal 600 mg/day and calcium dobesilate 500 mg twice daily. These medications were continued throughout the patient's ulcer-specific treatment.

Treatment of the ulcer itself was initiated by the patient's general practitioner with oral ciprofloxacin 500 mg every 12 hours for two months. As a result of bacterial susceptibility testing, three months later antibiotic treatment was continued with clavulanic acid plus amoxicillin (875 mg three times daily) for 15 days. The patient also received pentoxifylline (600 mg twice daily). General wound care was applied with weekly gentle mechanical and enzymatic debridement with Iruxol and Intrasite hydrogel. His response to this initial treatment was disappointing, and the heel ulcer remained at Wagner grade III (Figures 1C and 1D). The three decubital lesions remained at Wagner grade II. In parallel, his general condition deteriorated, and vascular surgery was contraindicated.

### Investigation

Given the lack of response, risk of amputation, and general deterioration in this patient's condition, bosentan was initiated on a compassionate use basis, with informed consent, three months after his hospitalization. Antibiotic therapy was discontinued and not reinstated during the course of treatment with bosentan. Bosentan has been shown to prevent the occurrence of new digital ulcers in patients with systemic sclerosis and a history of digital ulceration at a dose of 62.5 mg twice daily and titrated up to 125 mg twice daily after four weeks [9]. However, given our patient's age and history of cardiopathy, bosentan was initiated at a dose of 62.5 mg once daily for one week and titrated up to a maintenance dose of 62.5 mg every 12 hours twice daily thereafter. Following a two-week treatment period at the maintenance dose, all ulcers had improved and marked granulation tissue was apparent on the heel ulcer (Figure 1E) and the ulcer on the flexure of the right ankle. The sacral and external malleolar ulcers had both healed rapidly. The patient's general condition improved in parallel.

Following 21 weeks of bosentan treatment, the ulcer on the flexure of the right ankle and the heel ulcer had healed (Figure 1F), and the patient was able to walk using a walking aid. His bosentan therapy was well-tolerated. Monitoring of blood pressure, erythrocyte sedimentation rate, and alanine and aspartate aminotransferase levels during treatment showed no abnormalities. Bosentan was discontinued upon ulcer healing with no relapse observed to date.

## Discussion

The sequence of events observed in this patient suggests a beneficial role for dual endothelin receptor antagonism, as no other known or relevant therapeutic intervention was initiated concomitant to treatment with bosentan. However, as a single case report, several limitations warrant acknowledgement. Wound healing is influenced by multiple variables, and it was not possible to strictly control for all potential confounders in this case. In addition, there are limited data describing neurological and cardiovascular status (for example, extent of neuropathy, ankle-brachial index) available for this patient. Furthermore, the etiology of these ulcers appears intricate and may involve several pathological processes. For example, neuropathy was documented by electromyography and may be implicated in the exposure to heat, which was considered the cause of the heel ulcer. After the patient became unable to walk, the pressure that caused the decubital lesions most likely perpetuated all ulcers, and infection may have caused them, too. Macrovasculopathy is also a possible contributory factor to the lower-limb ulcers, and diabetic skin microangiopathy should be regarded as a common underlying cause for the appearance of all ulcers and their resistance to standard therapy. Although our patient had congestive heart failure and peripheral edema, these occurred six weeks after the appearance of the ulcer and, in our opinion, were not related to the development of his lesions.

Having acknowledged these limitations, we think that this case report adds to the increasing evidence in support of a key role for endothelin in the pathogenesis of diabetic macro- and microangiopathy. Endothelin plasma levels are elevated in patients with type 2 diabetes and correlate positively with diabetic vascular disorder [4,5], including diabetic skin microangiopathy, which leads to the development of chronic foot ulcers. Improvements in macro-vascular as well as microvascular functioning have been reported following the use of endothelin receptor antagonists in animal models [5] and in patients with diabetes [10-14], and endothelin receptor antagonists have been shown to improve the nutritive skin microcirculation. The observations in this case, especially given the timing of improvement in relation to bosentan administration, could be explained by the effect of bosentan on the diabetic skin microangiopathy, hence supporting a role for endothelin in its pathogenesis [4-6]. The efficacy of dual endothelin receptor antagonism has already been shown in other manifestations of diabetic microangiopathy [4,5]. Although unlikely, the effects of bosentan on glycemic control [14] and the wound-healing process [16] may also have contributed to our patient's outcome.

## Conclusions

While the observations reported here should be interpreted with caution and need to be confirmed in a controlled study, the sequence of events is suggestive of a beneficial role for bosentan in our patient. These findings are consistent with current knowledge on the role of endothelin in vascular complications of diabetes and support continued investigation of endothelin in the pathophysiology of untreatable skin ulcers as well as other manifestations of diabetic microangiopathy. This original case report will be of interest primarily to diabetologists and dermatologists.

## Consent

Written informed consent was obtained from the patient for publication of this case report and any accompanying images. A copy of the written consent is available for review by the Editor-in-Chief of this journal.

## Competing interests

We confirm that we have no financial or non-financial competing interests. We have not received funding in the form of a grant or received fees for the writing of this article. Actelion Spain paid for medical writing assistance, as we wanted the opportunity for our case to be published in an international journal. As non-native English speakers, the process is difficult, so we accepted the support of Elements Communications Ltd, funded by Actelion Spain, which we duly acknowledge as per Good Publications Practice.

## Authors' contributions

FAR diagnosed and treated the patient. CLG and MBS reviewed the case. All authors have read and approved the final manuscript.
